# Intestinal differentiation involves cleavage of histone H3 N-terminal tails by multiple proteases

**DOI:** 10.1093/nar/gkaa1228

**Published:** 2021-01-04

**Authors:** Karin Johanna Ferrari, Simona Amato, Roberta Noberini, Cecilia Toscani, Daniel Fernández-Pérez, Alessandra Rossi, Pasquale Conforti, Marika Zanotti, Tiziana Bonaldi, Simone Tamburri, Diego Pasini

**Affiliations:** IEO European Institute of Oncology IRCCS, Department of Experimental Oncology, Via Adamello 16, 20139 Milan, Italy; IEO European Institute of Oncology IRCCS, Department of Experimental Oncology, Via Adamello 16, 20139 Milan, Italy; IEO European Institute of Oncology IRCCS, Department of Experimental Oncology, Via Adamello 16, 20139 Milan, Italy; IEO European Institute of Oncology IRCCS, Department of Experimental Oncology, Via Adamello 16, 20139 Milan, Italy; University of Milan, Department of Health Sciences, Via A. di Rudinì, 8, 20142 Milan, Italy; IEO European Institute of Oncology IRCCS, Department of Experimental Oncology, Via Adamello 16, 20139 Milan, Italy; IEO European Institute of Oncology IRCCS, Department of Experimental Oncology, Via Adamello 16, 20139 Milan, Italy; IEO European Institute of Oncology IRCCS, Department of Experimental Oncology, Via Adamello 16, 20139 Milan, Italy; IEO European Institute of Oncology IRCCS, Department of Experimental Oncology, Via Adamello 16, 20139 Milan, Italy; IEO European Institute of Oncology IRCCS, Department of Experimental Oncology, Via Adamello 16, 20139 Milan, Italy; IEO European Institute of Oncology IRCCS, Department of Experimental Oncology, Via Adamello 16, 20139 Milan, Italy; University of Milan, Department of Health Sciences, Via A. di Rudinì, 8, 20142 Milan, Italy; IEO European Institute of Oncology IRCCS, Department of Experimental Oncology, Via Adamello 16, 20139 Milan, Italy; University of Milan, Department of Health Sciences, Via A. di Rudinì, 8, 20142 Milan, Italy

## Abstract

The proteolytic cleavage of histone tails, also termed histone clipping, has been described as a mechanism for permanent removal of post-translational modifications (PTMs) from histone proteins. Such activity has been ascribed to ensure regulatory function in key cellular processes such as differentiation, senescence and transcriptional control, for which different histone-specific proteases have been described. However, all these studies were exclusively performed using cell lines cultured *in vitro* and no clear evidence that histone clipping is regulated *in vivo* has been reported. Here we show that histone H3 N-terminal tails undergo extensive cleavage in the differentiated cells of the villi in mouse intestinal epithelium. Combining biochemical methods, 3D organoid cultures and *in vivo* approaches, we demonstrate that intestinal H3 clipping is the result of multiple proteolytic activities. We identified Trypsins and Cathepsin L as specific H3 tail proteases active in small intestinal differentiated cells and showed that their proteolytic activity is differentially affected by the PTM pattern of histone H3 tails. Together, our findings provide *in vivo* evidence of H3 tail proteolysis in mammalian tissues, directly linking H3 clipping to cell differentiation.

## INTRODUCTION

Histones are the principal protein components of chromatin. Their N-terminal regions protrude from the globular core of nucleosomes, are rich in lysine and arginine residues and serve as substrate for a complex pattern of regulatory post-translational modifications (PTMs) central to most biological processes (1), including a variety of human diseases like cancer, metabolic and neurological disorders, as well as inflammation (2).

Several studies reported that proteolytic cleavage of histones N-terminal tails is mediated by the activity of nuclear peptidases. This was termed clipping and is conserved across species from ciliates to mammalian cells. Histone H2A clipping activity was observed in the mid 70s in calf thymus chromatin (3) and considered a mechanism to facilitate transcription and replication (4). Clipping of the first six N-terminal residues of histone H3 was described in *Tetrahymena* a few years later and functionally linked to conjugation (5,6). Since then, the active enzymatic cleavage of the histones tails has been envisaged as a rapid way to change epigenetic settings upon specific stimuli (7).

H3 clipping has been described in different mammalian cell types such as embryonic stem cells (ESCs) (8,9), senescent fibroblasts and melanocytes (10), peripheral blood mononuclear cells (PBMCs) (11), Raji cells (12) and hepatocytes (13). All these studies were aimed to identify the enzymes, the cleavage sites or the cellular processes involved. Counterintuitively, the described endopeptidases were biochemically unrelated and were not part of the same protease family, suggesting high promiscuity for histone tail cleavage (14–16). In particular, H3 clipping has been frequently associated with cellular differentiation in several *in vitro* contexts. This included mouse and human ESC exposed to retinoic acid or osteoblasts treated with Receptor activator of nuclear factor kappa-B ligand (RANKL) (17). Each study identified several cleavage sites and different enzymes with clipping properties. The cysteine protease Cathepsin L was described for mESCs, while an undefined serine protease was associated with hESC. Clipping in osteoclastogenesis was linked to the extracellular matrix metalloprotease MMP-9 (8,9,17). Furthermore, Cathepsin L has been described to cleave the first 21 residues of the histone variant H3.3 during the senescence of human fibroblasts and melanocytes (10). Thus, histone H3 clipping seems to preferentially involve metallo, serine or cysteine proteases acting on the same substrate in specific contexts. More recently, the Jumonji C domain (JmjC) containing subfamily members JMJD5 and JMJD7 were also described to retain protease activities for arginine methylated histones tails (18,19).

The intestine is composed of a simple columnar epithelium that, leaning on lamina propria and muscularis mucosae, forms a continuous and relatively impermeable membrane. Its fast renewal rate sustains homeostasis in a highly challenging environment and relies on cell division, maturation, and migration sustained by proliferative stem cells (ISC) that reside at the bottom of epithelial invaginations called crypts of Lieberkühn (20,21). The main function of the small intestine, which is mainly composed of enterocytes, is to maximize absorption. Enterocytes differentiate by migrating away from the crypt bottom, populating the intestinal villi to create an absorptive surface for fluids and nutrients. At the tip of the villi, older cells die for apoptosis and shed off into the lumen.

Here, we report for the first time that the N-terminal tail of histone H3 undergoes extensive proteolytic cleavage specifically in the differentiated compartment (villi) of the mouse small intestinal tract. We demonstrate that Cathepsin L and Trypsins coordinate together H3 cleavage both *in vivo* and in 3D organoid cultures. These proteases showed a differential sensitivity for native nucleosomal substrates *in vitro* and played a role in organoid maturation. Overall, our findings uncover a mechanism for *in vivo* H3 clipping in mammalian adult tissue and suggest the involvement of the enzymatic removal of histone tails in the homeostasis of adult tissues.

## MATERIALS AND METHODS

### Animal procedures

Mice were housed according to the guidelines set out in Commission Recommendation 2007/526/EC, 18 June 2007. All experiments were performed in accordance with the Italian Laws (D.L.vo 116/92 and following additions), which enforces EU 86/609 Directive (Council Directive 86/609/EEC of 24 November 198).

### Tissue purification and 3D cultures

Small intestines were flushed in ice-cold PBS with P.I.C. (1:200, Protease Inhibitor Cocktail III, #539134, Calbiochem) and opened longitudinally. Villi were scraped using glass coverslips and collected in ice-cold PBS with P.I.C. Crypts-containing tissue was cut into small pieces and collected in ice-cold PBS. After two sequential incubations in cold PBS/EDTA (20 min in 1 mM EDTA and 45 min in 5 mM EDTA), crypts were released by mechanical disaggregation. In experiments with fixed and frozen samples, after animal death, organ withdrawn and fast flush of the intestinal lumen with cold PBS1/P.I.C., villi were scraped and fixed with 4% formaldehyde or flash-frozen into liquid nitrogen (1–2 min for each procedure). Both samples were then lysed in urea buffer (25 mM Tris pH 6.8, 6 M urea, 1 mM EDTA, 10% glycerol). Minigut cultures were performed according to (22). Briefly, purified crypts were plated 800 cells/40 μl Matrigel (BD Biosciences) in 24-well plates. Miniguts were cultured in Advanced DMEM/F12, supplemented with 10 mM HEPES, 2 mM GlutaMax, antibiotics, N2, retinoic acid-free B27 supplements (all from Invitrogen), 1.25 mM *N*-acetyl cysteine (Sigma), 50 ng/ml EGF (Preprotech), 100 ng/ml Noggin (Preprotech), mRSPO1 (homemade), recombinant Wnt3a (homemade), 10 μM Jagged1 peptide (AnaSpec), and 10 μM Y-27632 (Selleck Chemicals). Growth factors were added by replacing the medium daily. Protease inhibitors were added from the day after plating: E64 (1.2 μM final) and/or soybean trypsin inhibitor (SBTI) (1.2 mM final), or Z-VAD-fmk (10 μM and 20 μM).

### Cell extract preparation from villi

Total cell extracts were prepared in S300 buffer (20 mM Tris pH 8.0, 300 mM NaCl, 10% glycerol, 0.2% Igepal). After 20 min incubation on ice, following centrifugation (13 000 rpm, 10 min, 4°C), pellets containing endogenous histones were lysed in urea buffer (25 mM Tris pH 6.8, 6 M urea, 1 mM EDTA, 10% glycerol), sonicated and boiled immediately with SDS-Laemmli sample buffer. Cytosolic and nuclear extracts were prepared with Nuclear Prep buffer (10 mM Tris pH 8.0, 100 mM NaCl, 2 mM MgCl, 300 mM sucrose, 0.2% Igepal) on ice for 5 min. Nuclei were isolated by centrifugation (2500 rpm, 10 min, 4°C). Nuclei were washed once in Nuclear Prep buffer, lysed in S300 buffer, incubated on ice for 20 min and centrifuged for 10 min at 13 000 rpm at 4°C. Protease inhibitors were omitted when used for *in vitro* assays.

### Histone digestion for mass spectrometry

20 μg of chromatin bound fraction from intestinal villi were separated on a 17% SDS-PAGE gel. For in-gel digestions, bands corresponding to the full-length and clipped forms of histone H3 were separately excised, chemically acylated with propyonic anhydride and in-gel digested with trypsin. The digestion was performed as previously described (23), with the exception that the extraction of the digested peptides from the gel was performed with acetonitrile 50% and 100%. The samples were concentrated to a volume below 3 μl, diluted to 9 μl with water and derivatized with phenyl isocyanate for 1.5 h, using a protocol adapted from (24). After the digestion, the peptides were mixed with peptides derived from the digestion of heavy-labeled histone H3 (generated as previously described (23,25)), which was used as an internal standard for quantification. The samples were then desalted on handmade StageTips columns (23).

### LC–MS/MS analysis of histone PTMs

Peptide mixtures were separated by reversed-phase chromatography on an EASY-Spray column (Thermo Fisher Scientic), 25-cm long (inner diameter 75 μm, PepMap C18, 2 μm particles), which was connected online to a Q Exactive Plus instrument (Thermo Fisher Scientific) through an EASY-Spray™ Ion Source (Thermo Fisher Scientific). Solvent A was 0.1% formic acid (FA) in ddH2O and solvent B was 80% ACN plus 0.1% FA. Peptides were injected in an aqueous 1% TFA solution at a flow rate of 500 nl/min and were separated with a 50-min linear gradient of 10–45% of solvent B, at a flow rate of 250 nl/min. The Q Exactive HF instrument was operated in the data-dependent acquisition (DDA) mode to automatically switch between full scan MS and MS/MS acquisition. Survey full scan MS spectra (*m*/*z* 300–1350) were analyzed in the Orbitrap detector with a resolution of 70 000 at *m*/*z* 200. The 12 most intense peptide ions with charge states between 2 and 4 were sequentially isolated to a target value for MS1 of 3 × 10^6^ and fragmented by HCD with a normalized collision energy setting of 28%. The maximum allowed ion accumulation times were 20 ms for full scans and 80 ms for MS/MS, and the target value for MS/MS was set to 1 × 10^5^. The dynamic exclusion time was set to 20 s, and the standard mass spectrometric conditions for all experiments were as follows: spray voltage of 1.8 kV, no sheath and auxiliary gas flow.

### Histone PTM data analysis

The acquired RAW data were analyzed using Epiprofile 2.0 (26), selecting the SILAC option, or manually, as described (23). For each histone modified peptide, the % relative abundance (%RA) was estimated by dividing the area under the curve (AUC) of each modified peptide for the sum of the areas corresponding to all the observed forms of that peptide and multiplying by 100. Light/heavy (L/H) ratios of %RA were then calculated. The AUC values for all the samples analyzed are reported in Supplementary Table S1. The levels of H3.1/2 and H3.3 were calculated by using peptide 27–40, which differs in one amino acid in H3.3 and is thus distinguishable by MS. The sum of AUCs for all the differentially modified forms of peptides 27–40 in the light and heavy channels were used to calculate a L/H AUC ratio, which was then normalized over the full-length form of histone H3.1/2 or H3.3.

### Chromatin immunoprecipitation (ChIP)

ChIP experiments were performed according to standard protocols as described previously (27). Briefly, 1% formaldehyde cross-linked chromatin was sheared to 500–1000 bp fragments by sonication and incubated overnight in IP buffer (33 mM Tris–HCl pH 8, 100 mM NaCl, 5 mM EDTA, 0.2% NaN3, 0.33% SDS, 1.66% Triton X-100) at 4°C with the indicated antibodies (Supplementary Table S2). For histone modifications, 180 μg of chromatin supplemented with 5% spike-in of S2 Drosophila chromatin (prepared in the same manner) and 5 μg of antibodies were used. The next day, chromatin lysates were incubated for 3 h with protein-A Sepharose beads (GE Healthcare). Beads were washed 3 times with low-salt buffer (150 mM NaCl, 20 mM Tris–HCl pH 8, 2 mM EDTA, 0.1% SDS, 1% Triton X-100) and one time with high-salt buffer (500 mM NaCl, 20 mM Tris–HCl pH 8, 2 mM EDTA, 0.1% SDS, 1% Triton X-100), and then resuspended in decrosslinking solution (0.1 M NaHCO_3_, 1% SDS) overnight. DNA was purified with QIAquick PCR purification kit (QIAGEN) according to manufacturer's instructions. DNA libraries were prepared with 2–10 ng of DNA using an in-house protocol (28) by the IEO genomic facility and sequenced on an Illumina HiSeq 2000.

### ChIP-seq

Spiked-in ChIP-seq libraries (ChIP-Rx) were aligned to the mouse reference genome mm10 and fly reference dm6 using bowtie v1.1.2 (29) with parameters -k1 -I 10 -X 1000 –best. PCR duplicates were removed using samblaster (30). Ambiguous reads mapping to both mm10 and dm6 were discarded. For H3K27me3, H3K4me1, H3K4me3 and H3K9me3 peaks were called using MACS2 v2.1.1 (31) with parameters -f BAMPE–keep-dup all -m 10 30 -p 1e-10. To normalize for differences in sample library size, a scaling factor for each sample was calculated as described in (32) and was applied during BigWig file generation with the parameter –scaleFactors from bamCompare (deeptools 3.1) (33) with a bin size of 50. Deeptools 3.1 was also used to produce heatmaps with the functions computeMatrix and plotHeatmap. To create genome-wide scatter plots of ChIP-seq signal, the function multiBigwigSummary from deeptools 3.1 was used to create a matrix of 10 kb non-overlapping bins for every sample, using the bigwig files obtained with the procedure explained above.

### Nucleosome purification (MNase)

10^7^ 293T cells were washed in ice-cold PBS, resuspended in 5 ml buffer B (15 mM Tris pH 7.6, 15 mM NaCl, 60 mM KCl, 2 mM EDTA, 0.5 mM EGTA, 300 mM sucrose, 1 mM DTT, P.I.C.) and lysed adding 5 ml of lysis buffer (buffer B with 0.4% NP40). Samples were incubated 5 min on ice and nuclei collected by centrifugation (1500 rpm, 5 min, 4°C), washed twice in 10 ml buffer D (15 mM Tris pH 7.6, 15 mM NaCl, 60 mM KCl, 300 mM Sucrose), resuspended in MNase buffer (20 mM Tris pH 7.6, 5 mM CaCl_2_) and digested with 3 U/ml of MNase (Roche) at 37°C for 100 min. The reaction was stopped with EDTA (10 mM final). Nucleosomes were checked by Coomassie stained SDS-PAGE (17%) and agarose gel (1%).

### *In vitro* clipping assay

Extracts were incubated for 1–2 h at 37°C in S300 buffer with 350 nmol of nucleosomes in 30 μl total volume and analysed by immunoblotting. Protease inhibitors were added to the reaction before substrate addition (list available in Supplementary Table S2).

### Chromatography

Villi nuclei (15 mice) were resuspended in 10 ml of S300 buffer, incubated on ice for 20 min and clarified (13 000 rpm, 20 min, 4°C). Supernatant was filtered (0.45 μm), diluted 10× with a dilution buffer (20 mM Tris pH 8.0, 10% glycerol), and loaded on a Resource Q (GE Healthcare, 1 ml) linked in serie to a Resource S (GE Healthcare, 1 ml) equilibrated in Res buffer (20 mM Tris pH 8.0, 0.5 mM EDTA, 30 mM NaCl, 1 mM DTT). Columns were eluted separately with 75, 150, 225, 300, 400, 500 mM NaCl Res buffer over 30 CV in total. 20 μl of each fraction were used for *in vitro* assay.

### Immunopurification and depletions

500 μg of villi nuclear extracts were incubated with 3 μg of antibodies overnight at 4°C on a rotating wheel. Antibodies were recovered with protein G or A sepharose beads for 2 h at 4°C. After retrieval of the not bound fraction, beads were washed 3–5 times in S300 buffer and boiled in SDS-Laemmli.

### Immunofluorescence

Mouse small intestine was collected and fixed with 4% paraformaldehyde (PFA) for 2 h at 4°C. The tissue was then cryopreserved in 30% sucrose overnight, embedded in O.C.T. (Thermo Scientific 12678646) and cut at 5 μm in thickness. The tissue sections were incubated in PBS containing 0.5% Triton X-100 with 1% BSA and 10% normal goat serum (1 h at RT) to block nonspecific antibodies. Slides were then incubated with H3K14ac specific antibody (1:200 dilution) overnight at 4°C, washed in PBS and incubated for 1 h at room temperature in the dark with secondary fluorescent antibody Alexa Fluor 488 (Jackson ImmunoResearch). Cell nuclei were stained with 4,6-diamidino-2-phenylindole (DAPI, 1:3000, Sigma Aldrich), and finally sealed with Mowiol 4-88 (Sigma Aldrich). Images were taken with Leica DM6B microscope.

### Antibodies

The list of antibodies is available in Supplementary Table S2.

## RESULTS AND DISCUSSION

### Intestinal villi present extensive histone N-terminal tail clipping *in vivo*

**Figure 1.**
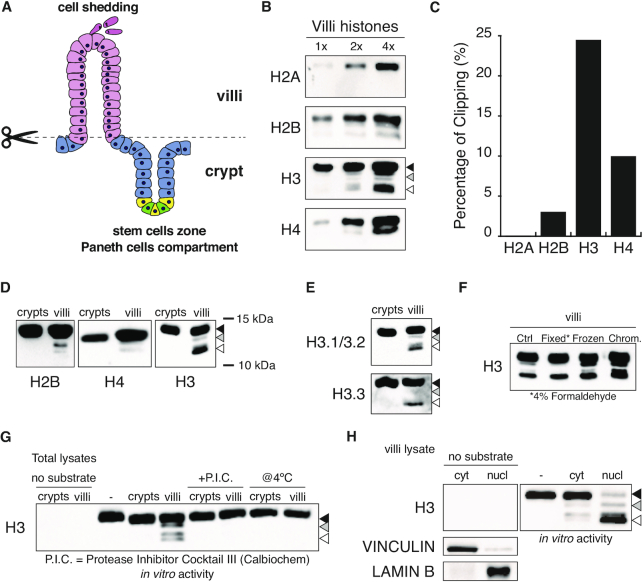
Histones N-terminal tails are cleaved in the differentiated cells of the mouse intestine. (**A**) Schematic representation of the intestinal epithelium architecture. In pink are depicted the villi enterocytes, in blue the crypts cells, in green the intestinal stem cells (ISCs), in yellow, Paneth cells. (**B**) Western blot analysis of histones extracted from the villi fraction of the intestinal epithelium. Three dilutions were loaded on SDS-PAGE gel to gauge the linearity of the antibodies. The arrow heads indicate the full length (black), intermediate (gray) and fully truncated (white) histone H3 forms. (**C**) Percentage of the truncated forms relative to the full length counterpart for each histone calculated by intensity of the western blot analysis shown in (B). (**D**) Western blot analysis of endogenous histone H2B, H4 and H3 extracted from intestinal crypt or villi. (**E**) Western blot analysis of endogenous histone H3 variants, H3.1/3.2 and H3.3, extracted from intestinal crypt or villi. (**F**) Western blot analysis of histone H3 on total extracts from normal (Ctrl), previously fixed in 4% formaldehyde (Fixed), flash frozen (Frozen) purified villi cells and villi chromatin bound fraction (Chrom.). (**G**) Clipping *in vitro* assay performed on nucleosomes purified from 293T cells. 30 ug of crypt or villi total extract were incubated with unclipped nucleosomes for 2 h at 4 or 37°C. Protease Inhibitor Cocktail III from Calbiochem (P.I.C.) actively inhibits histone H3 clipping at 37°C when added to the reaction mix. (**H**) Clipping *in vitro* assay performed as in (G) using equal ratio of cytosolic or nuclear villi extracts. Vinculin and lamin B were used as fractionation controls.

Histones isolated from intestinal villi (Figure 1A) showed faster migrating bands when immunoblotted with antibodies recognizing the C-terminal portion of the four different histone proteins (Figure 1B). This suggests that different histones undergo N-terminal proteolytic cleavage (clipping) in the differentiated fraction of the intestinal epithelium. Among them, while histone H2A did not present any cleaved forms (Figure 1B and C), histone H3 resulted to be the most affected (∼25%, Figure 1C).

We tested whether proteolytic histone cleavage was specific for the villi fraction. Western blot analysis on crypts and villi extracts demonstrated that clipping of H3, H2B and H4 was restricted to the villi compartment, with H3 clipping being highly detectable with the H3.1/.2 or H3.3 variants being similarly affected by this proteolysis (Figure 1D and E). Based on the size of the fastest migrating band, we estimated that the H3 tail loses approximately up to 20 N-terminal residues (Figure 1D, F and Supplementary Figure S1A). The intestinal lumen is rich in proteases and it is well known that the time from explant affects cell death which gradually expands from the tip to the bottom of the villi (34). To exclude that proteolysis could be a consequence of sample manipulation, we either fixed with formaldehyde or flash-froze into liquid nitrogen the isolated villi immediately upon explant to inactivate any enzymatic activity. Western blot analyses showed that H3 proteolytic cleavage was fully preserved in these conditions (Figure 1F), demonstrating that H3 clipping occurs *in vivo* in the villi differentiated compartment.

To further characterize such activity, we have set up an *in vitro* assay in which native nucleosomes were incubated with protein extracts of different sources. Using villi vs. crypt extracts, we demonstrated that the proteolytic activity was present specifically in the differentiated portion of the small intestine (Figure 1G). Moreover, the subcellular fractionation of enterocytes (the most abundant cell type of villi) demonstrated that H3 proteolytic activity is preferentially localized in the nucleus (Figure 1H). No nuclear proteolytic activity was observed on either H2B or H4 substrates (Supplementary Figure S1B) suggesting that H3 is the preferential substrate for intestinal histone N-terminal proteolysis. Overall, these results demonstrate that *in vivo* H3 N-terminal truncation is catalysed by villi-specific nuclear proteolysis.

### Histone H3 clipping is coordinated by multiple activities able to sense the chromatin environment

**Figure 2.**
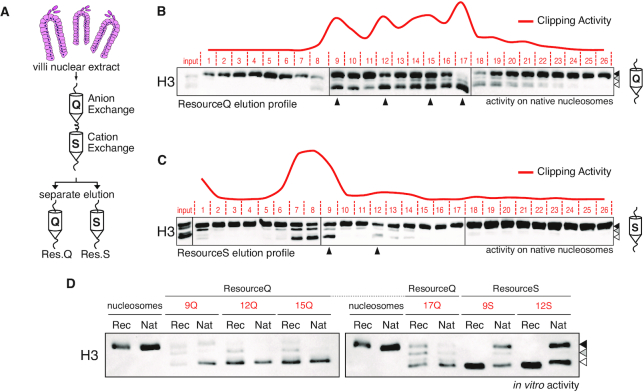
Histone H3 clipping in intestinal villi involves multiple proteases. (**A**) Schematic representation of the purification protocol. (**B, C**) Elution profiles of the quantified *in vitro* activity obtained by incubating the same amount of anion (B) and cation (C) exchange fractions with unclipped native nucleosomes for 2 h at 37°C (red curve). Arrow heads indicated fractions used in further reactions. (**D**) Clipping activities of chosen fractions (arrowheads in B and C) on recombinant or native nucleosomal substrates.

**Figure 3.**
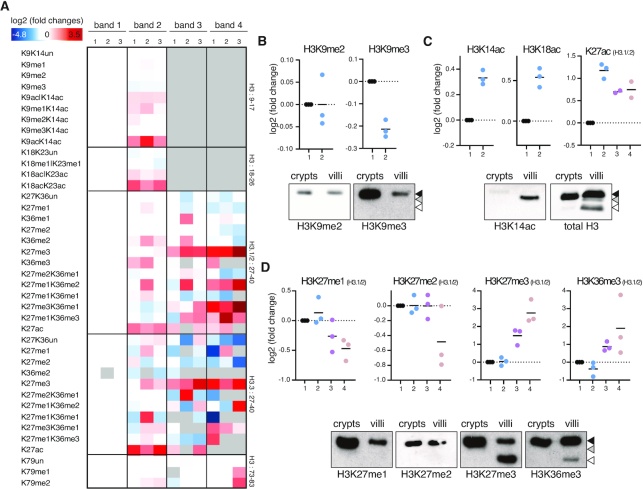
Mass Spectrometry analysis of histone H3 clipping in villi. (**A**) Heatmap display of histone PTM levels in the full-length (band 1) and clipped (bands 2–4, see Supplementary Figure S2A) forms of histone H3. L/H (light/heavy) relative abundances ratios were obtained using a spike-in strategy (light channel: sample, heavy channel: spike-in standard), and were normalized over the PTM levels in full-length histone H3. The gray color indicates peptides that were not quantified. 1, 2, 3: biological replicates. (**B–D**) Interleaved scatter plot display of the data shown in A for selected PTMs and Western blot analyses of crypts and villi histone H3 with the indicated antibodies, specific for H3 PTMs.

To characterize this activity, we set up a tandem purification system based on ion exchange chromatography to fractionate the villi nuclear extracts (Figure 2A). Nuclear extracts were loaded on anion and cation exchange resins sequentially (Figure 2A). Loaded columns were eluted individually applying a similar gradient of increasing ionic strength and tested for *in vitro* activity on native nucleosomes. Distinct peaks of proteolytic activity were eluted from both resins, suggesting that multiple activities could be involved in H3 clipping (Figure 2B and C). Representative fractions were selected and their activity tested on native vs. recombinant nucleosomes. While anion exchange (Res Q) active fractions were able to cleave H3 from native and recombinant nucleosomes to a similar extent, fractions selected from the cation exchange column (Res S) were less efficient in processing the native nucleosomal substrate (Figure 2D). This suggests that the eluted activities have distinct properties that could result in different sensitivities, for instance in the pattern of histone post-translational modifications (PTMs). Accordingly, we performed a mass spectrometry (MS) analysis of H3 modifications on histones extracted from villi, revealing selectivity for specific histone PTMs *in vivo* (Figure 3A, Supplementary Figures S2A, S2B and Table S1). While the H3:3–8 peptide (containing H3K4 and its modifications) was excluded from the analysis as intrinsically challenging for reliable MS detection, we found that other N-terminal PTMs (H3K9me2/3) were less abundant in the full length H3 with respect to its truncated form, echoing the loss of the N-terminal tail itself (Figure 3B). Acetylations of H3 appeared to be more abundant on the truncated forms of H3 (Figure 3C). Western blot analyses validated these results revealing that H3K14ac was only present in the intermediate H3 truncations but not detected neither on the full length H3 of villi nor on histone H3 purified from crypts (Figure 3C). This result suggests a mechanism in which proteolytically cleaved histone tail may stimulate *de novo* deposition of PTMs, creating a direct crosstalk between histone clipping and PTMs deposition. Alternatively, certain PTMs may foster the proteolytic processing of H3 and give rise to a feed-forward regulatory loop.

Finally, different H3K27 methylation forms showed distinct patterns. While mono- and di-methylations were enriched on the full length H3, H3K27me3 showed an opposite behaviour being more abundant on the truncated forms, similar to H3K36me3 distribution observed by MS (Figure 3D, Supplementary Figures S2C and D). Importantly, these effects occurred with the same efficiency on different histone H3 variants (Supplementary Figures S2B and D). Altogether these observations point to the existence of multiple endopeptidases that can target histone H3 for proteolytic N-terminal cleavage with the ability to sense the modification status of nucleosomes.

**Figure 4.**
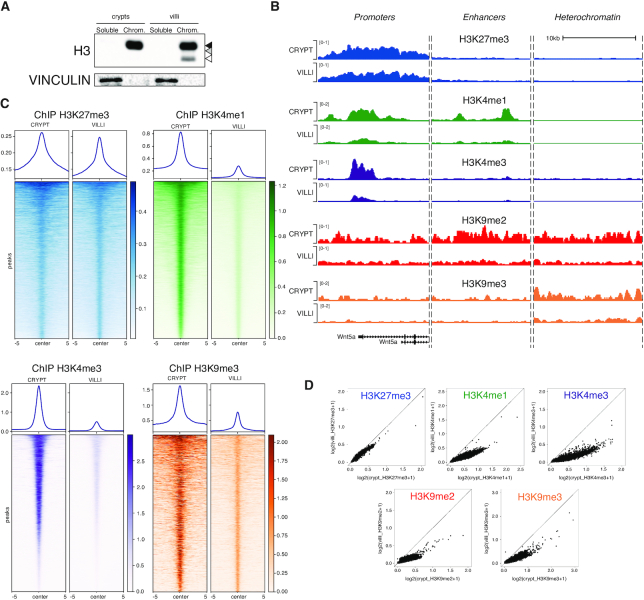
Genomic distribution of histone H3 modifications in crypts and villi. (**A**) Western blot analysis of endogenous histone H3 in soluble and chromatin bound fraction of crypts and villi cells. (**B**) Representative genomic snapshots of H3K27me3 (blue), H3K4me1 (green), H3K4me3 (purple) H3K9me2 (red) and H3K9me3 (orange) normalized ChIP-seq intensities across the Wnt5a gene locus, a representative enhancer and an heterochromatin region. (**C**) Heatmaps representing H3K27me3 (blue), H3K4me1 (green), H3K4me3 (purple) and H3K9me3 (orange) normalized ChIP-seq intensities across their corresponding peaks in villi samples. A genomic window of ±5 kb around the peak center is represented. (**D**) Scatter plots of H3K27me3 (blue), H3K4me1 (green), H3K4me3 (purple) and H3K9me3 (orange) genome-wide normalized ChIP-seq intensities. Each dot represents a 10 kb non-overlapping genomic window.

Since the truncated forms of histone H3 are fully retained into chromatin (Figure 4A), we performed ChIP-RX sequencing analyses in crypts and villi to obtain a genome-wide view of clipped PTMs. We selected H3 PTMs with specific functional properties and in which clipping was distinctly regulated (Figure 3). This included H3K27me3 and H3K4me3, which decorate promoter regions of repressed and active genes respectively; H3K4me1 which is enriched at enhancers; H3K9me2 with a broad genomic distribution and H3K9me3, which is enriched at heterochromatin sites (Figure 4B). As expected the different PTMs were specifically modulated in their localization across the crypts-villi axes that are consistent with changes in cell type composition (Supplementary Figure S3). H3K4me3 deposition remained essentially identical between the two compartments, in agreement with its role in marking both repressed (bivalent) and active promoters. H3K9me3 instead showed the largest degree of variation consistent with its role in heterochromatin formation, thus highlighting a reorganization of chromatin between these compartments. H3K27me3 presented instead a villi-specific accumulation of peaks suggesting that more differentiated compartments reduces their transcriptional plasticity. Consistent with our data, while H3K27me3 was retained at repressed promoters, the intensity for all other histone PTMs close to the N-terminus of the histone tail were drastically decreased in villi at their specific deposition sites (Figure 4B–D), thus reflecting their loss due to protease cleavage. Overall, this demonstrates that PTMs deposition remains modulated in relation to the functional cellular state and that H3 clipping can be a mechanism to decrease the local concentration of specific histone modifications residing in the most N-terminal portion of the histone tail without affecting their ‘cell type’ specific localization.

### Histone H3 tail cleavage is coordinated by both cysteine and serine proteases

**Figure 5.**
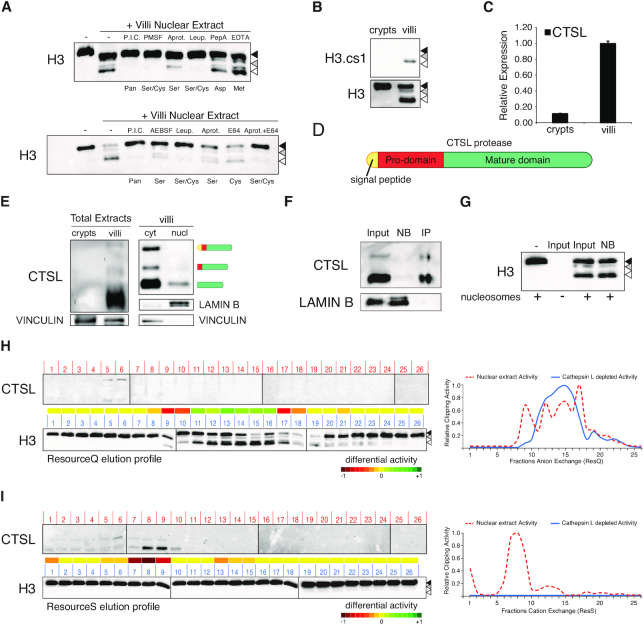
Cathepsin L is a cysteine protease involved in histone H3 clipping. (**A**) Clipping *in vitro* assay performed on nucleosomes purified from 293T cells using 10 ug of villi nuclear extract with or without protease inhibitors: Protease inhibitor cocktail III (P.I.C.), PMSF, AEBSF, Leupeptin (Leup.), Pepstatin A (PepA), EDTA, AEBSF, Aprotinin (Aprot.), E64 or a combination of these latter (Aprot.+E64). The labels below the blot indicate the specificity of the used inhibitors: all proteases (All); serine proteases (Ser), cysteine proteases (Cys), both (Ser/Cys), metallo-proteases (Met.), aspartic proteases (Asp). (**B**) Western blot analysis of endogenous histone H3 and H3.cs1 truncation extracted from intestinal crypt or villi. (**C**) Expression levels of CTSL in crypt and villi cells. Data represent mean ± SEM. (**D**) Scheme of Cathepsin L protein (CTSL). (**E**) Western blot analysis of CTSL in crypt and villi total extracts and in villi cellular compartments. (**F**) Western blot analysis of CTSL immunodepletion from villi nuclear extract. Lamin B was used as loading control (not bound, NB). (**G**) Clipping *in vitro* assay performed on nucleosomes purified from 293T cells using the same amounts of villi nuclear extract (input) and immunodepleted fraction (not bound, NB). (**H, I**) Elution profiles of CTSL and quantified *in vitro* activity obtained by incubating same amount of fractions eluted from anion (ResourceQ, H) or cation (ResourceS, I) exchange with unclipped nucleosomes for 2 h at 37°C. Heatmaps represent the differential activity with and without Cathepsin L (immunodepletion purifications vs nuclear extract purifications). Relative clipping activities on anion (right upper graph) or cation exchange fractions (right lower graph) purified from villi nuclear extracts (dashed red curves, as depicted in (Figure 2B and C)) and from villi nuclear extracts immunodepleted for Cathepsin L (blue curves).

In order to identify histone H3 proteolytic activities, we selected different protease inhibitors covering the same spectrum and concentrations of the inhibitor cocktail (P.I.C) used *in vitro* (Figure 1G). This included PMSF and Leupeptin to inhibit serine and cysteine proteases (35–38); Pepstatin A (PepA) for aspartic proteases (39) and EDTA for metallo-proteases. Aprotinin and AEBSF were used as serine specific protease inhibitors (36,40), while E64 was included to specifically inhibit cysteine proteases (41) (Supplementary Figure S4A). While EDTA and PepA had no effect, general serine/cysteine protease inhibitors fully abrogated H3 clipping (Figure 5A). Importantly, the pan-caspase inhibitor, Z-VAD-fmk (42), did not exert any inhibitory effect (Supplementary Figures S4B and C) excluding a contribution of Caspases activation and thus apoptosis in the proteolytic processing of H3 in the intestinal epithelium. The separate use of Aprotinin and E64 only showed a partial inhibition, however, their combined treatment fully inhibited proteolysis. Consistent with the MS data, no evident specificity towards particular H3 variants were observed (Supplementary Figure S4D). Together, this demonstrates that the combined activity of serine and cysteine proteases regulates the processing of histone H3 tails in intestinal villi.

### Cathepsin L is a cysteine protease involved in histone H3 tail clipping *in vivo*

The cysteine-protease Cathepsin L (CTSL) has been shown to retain proteolytic activity on histone H3 in other cellular contexts cleaving at residue A21. This creates a specific epitope called clipping site 1 (cs1) recognized by the H3.cs1 antibody (8). We detected cs1 among the endogenous truncated forms of H3 from villi (Figure 5B). Interestingly, H3.cs1 corresponded to an intermediate H3 truncation that was not the most abundant form. Consistently, this H3 truncation is formed *in vitro* by a villi-specific nuclear activity that was strongly inhibited by the cysteine protease inhibitor E64 (Supplementary Figure S4E). Overall, this suggests a direct involvement of CTSL in the villi-specific H3 proteolytic processing. CTSL full-length (inactive) and its processed (active) forms were found expressed in villi and also localized in the nucleus (Figure 5C–E). Thus, we immunodepleted CTSL from villi nuclear extracts using specific antibodies (Figure 5F) and tested the residual activity. Consistent with the contribution of distinct proteolytic activities, the CTSL-depleted nuclear lysate preserved efficient H3 cleavage also in the absence of CTSL (Figure 5G). However, to discriminate between the two putative activities with distinct elution properties, we further fractionated the CTSL-depleted lysate by ion exchange and found that while the Res Q activity was unaffected, the Res S activity was fully abrogated in absence of CTSL. Consistently, CTSL co-eluted specifically in the most active Res S fractions (Figure 5H and I), demonstrating that CTSL is one of the cysteine proteases responsible for histone H3 proteolytic cleavage in intestinal villi.

### The trypsin family of serine proteases are involved in histone H3 tail clipping in the intestinal villi

**Figure 6.**
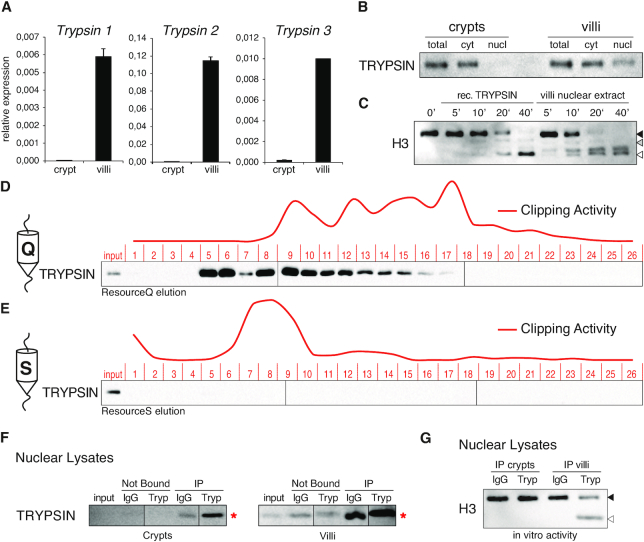
Trypsin is the serine protease involved in histone H3 clipping. (**A**) Expression levels of Trypsin 1, 2 and 3 in crypt and villi cells. Data represent mean ± SEM. (**B**) Western blot analysis of crypt and villi cellular compartments for trypsin localization. (**C**) Clipping *in vitro* assay using recombinant Trypsin or villi nuclear extract as enzymatic source. (**D, E**) Elution profiles of Trypsins from nuclear villi extract purification. Quantified *in vitro* activity of fractions was depicted as a red curve (see Figure 2). (**F**) Western blot analysis of Trypsin immunodepletion from crypts and villi nuclear extracts after 3 sequential rounds of immunopurification. Red asterisks indicate IgG light chains. (**G**) Clipping *in vitro* assay using immunoprecipitated Trypsins from crypt or villi nuclear extracts as enzymatic source. Mouse IgG were used as negative control.

Trypsins are serine proteases produced and secreted in their inactive trypsinogen forms by pancreatic acinar cells, and released into the intestinal lumen at the duodenum level (43). Trypsins can also be expressed by other cell types including cells of the small intestinal tract (44). We found that villi transcribe high levels of different trypsin genes (PRSS1–3; Figure 6A) and that Trypsins are expressed in both crypts and villi (Figure 6B). Importantly, only villi showed nuclear Trypsin localization in agreement with the villi-specific proteolytic cleavage of histone H3 tails (Figure 6B). Time-dependent incubation of nucleosomal substrates with recombinant Trypsin generated an analogous pattern of N-terminal truncations (Figure 6C). Importantly, Trypsins eluted specifically on the ion-exchange Res Q with a bimodal profile, with one of the two elution peaks corresponding to high H3 proteolytic activity (Figure 6D and E). Finally, although Trypsins immunodepletion from villi nuclear extracts was not efficient (Figure 6F), the incubation of immunoprecipitates with native nucleosomes resulted in specific cleavage of histone H3 tails (Figure 6G). Together, these results indicate that intestinal-expressed Trypsins are the serine proteases involved in H3 proteolytic cleavage in differentiated villi.

### Histone H3 clipping regulates intestinal organoid homeostasis

**Figure 7.**
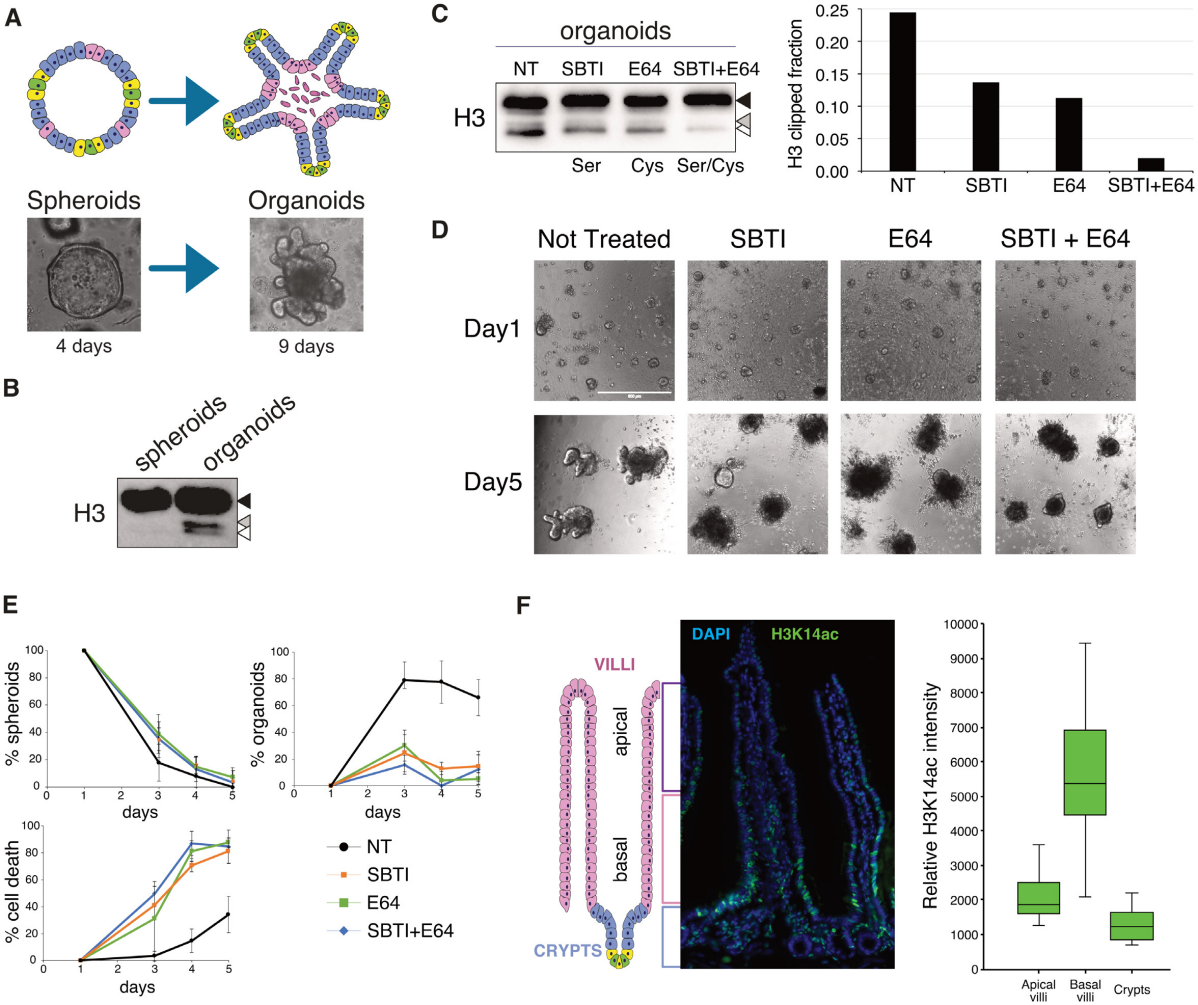
Histone H3 clipping is involved with intestinal organoids maturation. (**A**) Schematic representation and pictures of organoids cultured for 4 (spheroids) and 9 days (organoids). (**B**) Western blot analysis of histone H3 extracted from spheroids (3 days) and organoids (6 days). (**C**) Western blot analysis of histone H3 extracted from organoids cultured for 6 days in absence (not treated, NT) or in presence of Trypsins inhibitor (Soybean Trypsin Inhibitor, SBTI), cysteine inhibitor (E64) or a combination of both (SBTI + E64). Right graph represents the ratio of H3 clipped form over the full length in the four growth conditions. (**D**) Pictures of the treated organoids as in (C) at 1 and 5 days of growth. (**E**) % spheroids, % organoids and % cell death observed in the four conditions of the experiment in (C) and (D). (**F**) Immunofluorescence staining of H3K14ac (green) in the duodenum epithelial tissue. The DAPI (blue) staining represents the nucleus (magnification 40x). Box plots of normalized H3K14ac signal intensities along the crypt-villus axis (*N* = 8).

To provide further evidence of direct H3 clipping in villi, we established mouse intestinal organoid (or minigut) cultures starting from single stem cells (45). We used single cells directly derived from purified crypts, which did not display neither clipped histone H3 *in vivo* nor clipping activity *in vitro* (Figure 1D and G). Only ISCs will give rise to 3D organoids under these culture conditions (45). Miniguts first acquire a spheroidal structure composed of ISCs and intestinal progenitor cells (46) to later develop into budded-shaped structures (referred to as organoids) comprising crypt-like protrusions and villi-like internal patches of differentiated cells that recapitulate the architecture and composition of the intestinal tissue (Figure 7A). Consistent with our results, fully grown organoids displayed faster migrating H3 bands with a molecular weight comparable to the truncated forms found *in vivo* (Figure 7B and Supplementary Figure S5A). This confirmed that also in 3D organoid cultures, histone H3 underwent specific N-terminal cleavage (Supplementary Figure S5A). Importantly, similar to villi vs. crypt preparations, undifferentiated spheroid cultures (enriched of progenitors) showed no H3 clipping recapitulating the *in vivo* context. Consistent with our *in vitro* data (Supplementary Figures S4B and C), treatment of organoids with the pan-caspase inhibitor Z-VAD-fmk showed no inhibitory effect on H3 clipping, excluding the involvement of apoptosis also in this experimental setup (Supplementary Figures S5B and S5C).

The separate exposure of intestinal organoids to the soybean trypsin inhibitor (SBTI) or to the cysteine protease inhibitor E64 only partially affected the levels of the clipped H3. Importantly, when SBTI and E64 were combined, H3 N-terminal clipping in organoids was further reduced (Figure 7C), thus confirming the existence of the same enzymatic activities also in intestinal organoid cultures. Importantly, SBTI and E64 treatment affected organoid growth and morphology. The transition from spheroids to fully differentiated organoids with crypt-like protrusions was clearly delayed (Figure 7D), leading to a higher level of organoid mortality (% cell death in Figure 7E). Together, these results suggest that inhibition of the proteases involved in histone H3 clipping interferes with spherical mini-guts maturation leading to their complete collapse (Figure 7D and E). Whether the observed defects in organoid growth and morphology are the direct consequence of the loss of H3 clipping remains to be investigated.

Finally, we performed immunofluorescence staining with a H3K14ac specific antibody on small intestinal sections. H3K14ac was specifically detected on an intermediate H3 truncated form in villi by western blot and MS analyses (Figure 3A and C). Indeed, H3K14ac was absent in the crypt but specifically present in the basal region of the differentiated villi and disappeared while cells moved towards the tip (Figure 7F and Supplementary Figure S5D). This result strengthens a model that links H3 clipping to *in vivo* differentiation of intestinal cells, opening to the possibility of a step-wise proteolytic process occurring along the villus, from its proximal to distal part (tip).

These findings provide *in vivo* relevance to previous reports that used cell culture models to associate histone H3 clipping with differentiation and ageing (8,10,17). Previous studies have identified distinct endopeptidases able to cleave the histone H3 tail in different biological contexts (8,17,47), raising questions on how H3 clipping is regulated. Our data show that multiple activities can target H3 in the same context providing a new perspective on a combined action that takes advantage of biochemically distinct activities.

Our data highlight also a potential greater sensitivity of Cathepsin L for native nucleosomes, opening to the possibility that histone tails PTM patterns could modulate its proteolytic activity. Previous structural studies of Cathepsin L in a complex with a histone H3 tail peptide did not highlight a particular influence of specific PTMs in cleavage (48). However, these experiments did not assess PTM-dependent modulation of Cathepsin L activity within a nucleosomal context or chromatin organization. The selectivity of PTMs on truncated forms of H3 in villi further suggests that cleavage could be non-homogenous within the same cells and guided by the chromatin environment. Although it is possible that histone tail removal could serve as a rapid mechanism to erase epigenetic modifications at specific sites, the promiscuity of the enzymes involved and the abundance of *in vivo* cleavage rather suggest a broader role in modifying general chromatin structure. Major chromatin changes usually imply repositioning, partial or complete eviction of nucleosomes or changes in histone variant compositions of the core octamer (49). Altering chromatin dynamics can maintain the active or silent state of targeted genomic regions (50). Interestingly, SWI/SNF complexes have been reported to remodel human tailless nucleosomes with lower efficiency (51). Moreover, even inter-nucleosomal interactions were found to be destabilized when histones lack their tails, suggesting a possible role for histone clipping in modulating chromatin 3D structure and/or the association of specific factors (52). Lately, H3 tails and their acetylation states on chromatin have been shown to influence histone H1 CTD condensation (53). It is therefore possible to envision histone clipping as an epigenetic irreversible modification that could impact on chromatin structure altering histones/DNA contacts during the differentiation towards specialized post-mitotic cells as it occurs in intestinal villi development.

Overall, our data propose a model where the activity of Cathepsin L and Trypsins can shape the nucleosome structure by coordinating histone tail cleavage during the maturation of ISC towards fully differentiated intestinal cells. Therefore, the development of more sophisticated models directly targeting histone clipping in this context will be required to fully elucidate the role of H3 processing in intestinal development.

## DATA AVAILABILITY

The mass spectrometry data have been deposited to the ProteomeXchange Consortium (54) via the PRIDE partner repository with the dataset identifier PXD022171. Crypts and villi H3K4me1, H3K4me3, H3K9me2, H3K9me3 and H3K27me3 datasets are available on Gene Expression Omnibus under the accession number GSE 160776.

## Supplementary Material

gkaa1228_Supplemental_Files
