# Supplementary material for: Intestinal differentiation involves cleavage of histone H3 N-terminal tails by multiple proteases
Source: Nucleic Acids Res. 2021 Jan 4;49(2):791–804. doi: 10.1093/nar/gkaa1228 (PMC7826276; doi:10.1093/nar/gkaa1228)
Supplement: gkaa1228_Supplemental_Files [file gkaa1228_supplemental_files.zip › Ferrari_Supplementary info revised2.pdf]

Intestinal Differentiation Involves Cleavage of Histone H3 N-Terminal Tails by Multiple Proteases

SUPPLEMENTARY MATERIAL

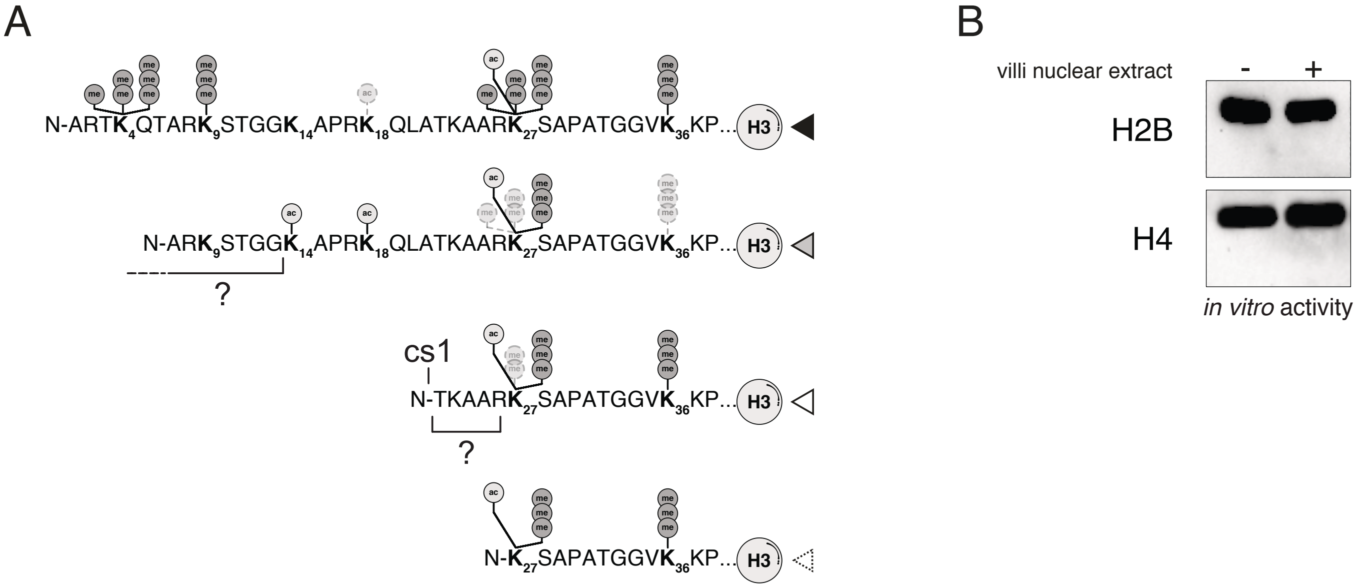
A
N-ARTK4QTARK9STGGK14APRK18QLATKAARK27SAPATGGVK36KP... H3
N-ARK9STGGK14APRK18QLATKAARK27SAPATGGVK36KP... H3
?
cs1
N-TKAARK27SAPATGGVK36KP... H3
?
N-K27SAPATGGVK36KP... H3
me
ac
B
villi nuclear extract
-
+
H2B
H4
in vitro activity

Supplementary Figure S1. Histones N-terminal tails are cleaved in the differentiated cells of the mouse intestine, related to Figure 1. (A) Schematic representation of histone H3 and its hypothesized forms based on Western blot results (arrow heads as in Figure 1B). Square brackets indicate potential cleavage sites (?) as determined by immunoblotting. The solid line marks the distal cleavage site (cs1). (B) Western blot analysis of H2B or H4 on clipping *in vitro* assay performed as in Figure 1G using nuclear villi extracts.

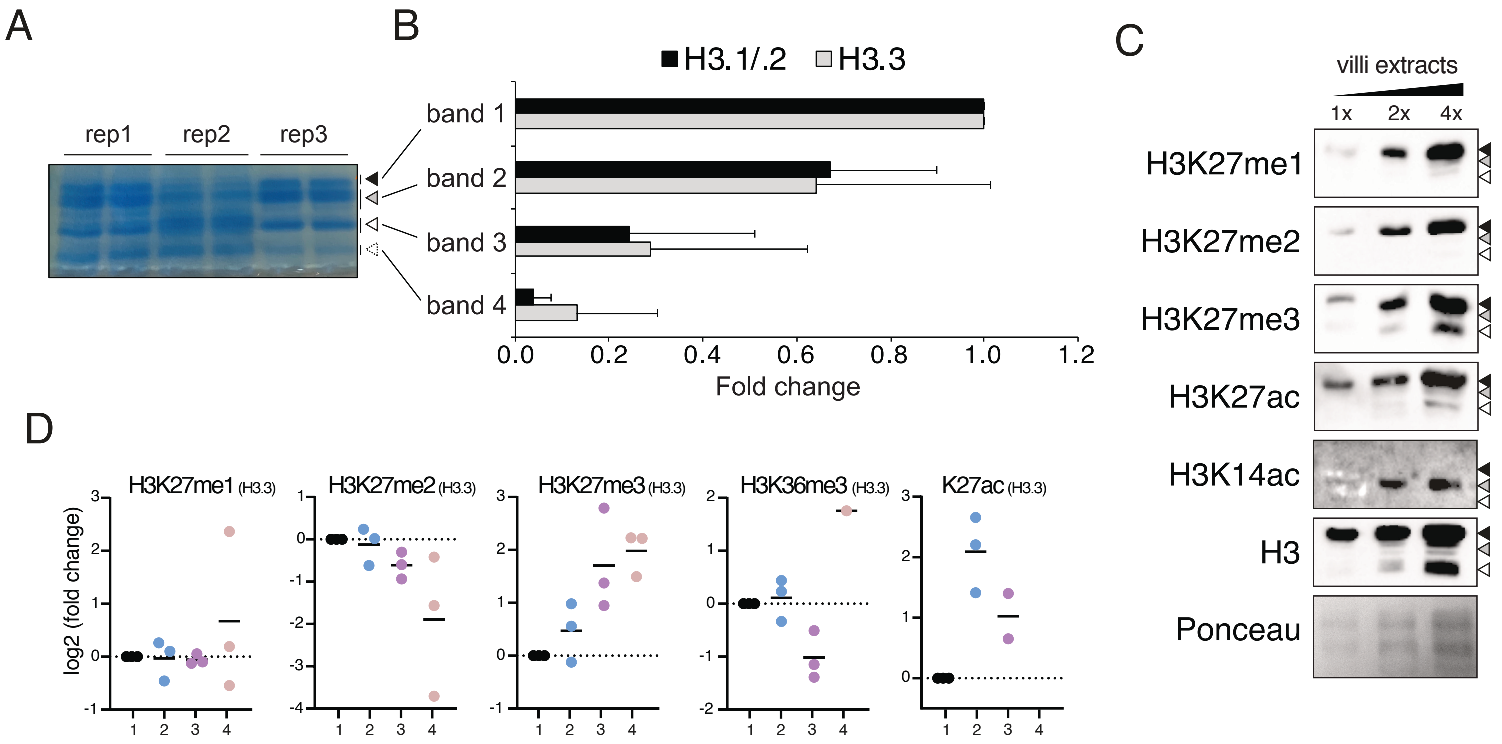
A
rep1
rep2
rep3
B
H3.1/.2
H3.3
band 1
band 2
band 3
band 4
0.0
0.2
0.4
0.6
0.8
1.0
1.2
Fold change
C
villi extracts
1x
2x
4x
H3K27me1
H3K27me2
H3K27me3
H3K27ac
H3K14ac
H3
Ponceau
D
log2 (fold change)
H3K27me1 (H3.3)
H3K27me2 (H3.3)
H3K27me3 (H3.3)
H3K36me3 (H3.3)
K27ac (H3.3)
1
2
3
4

Supplementary Figure S2. Mass Spectrometry analysis of histone H3 clipping in villi, related to Figure 3. (A) Coomassie staining of histones from villi. Vertical lines indicate the gel cut areas corresponding to analysed bands. (B) Histone H3.1/2 and H3.3 levels in full-length (band 1, panel A) and clipped (bands 2-4, panel A) histone forms. These levels were calculated by using peptide 27-40, which differs in one amino acid in histone H3.1/2 and H3.3 and is thus distinguishable by MS. Error bars show the standard deviation from three biological replicates. (C) Western blot analyses of villi histone H3 with the indicated antibodies, specific for H3 PTMs. (D) Interleaved scatter plot display of the data shown in Figure 3A for selected histone H3.3 PTMs.

A

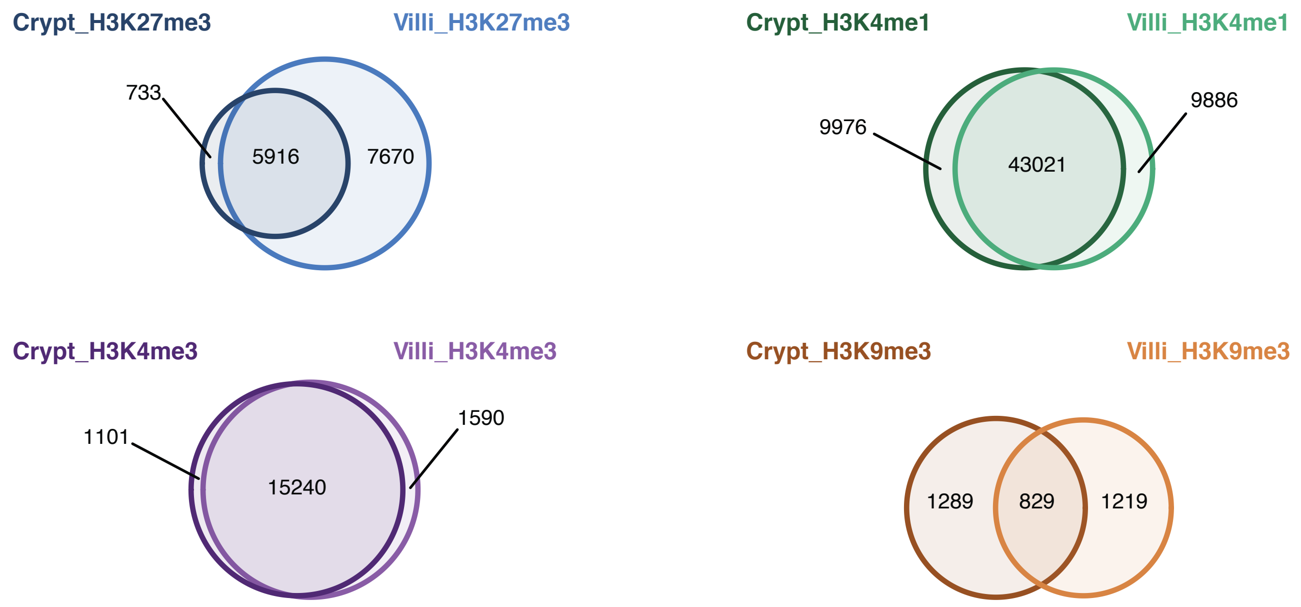
Crypt_H3K27me3
Villi_H3K27me3
733
5916
7670
Crypt_H3K4me1
Villi_H3K4me1
9976
43021
9886
Crypt_H3K4me3
Villi_H3K4me3
1101
15240
1590
Crypt_H3K9me3
Villi_H3K9me3
1289
829
1219

Supplementary Figure S3. Genomic distribution of histone H3 modifications in crypts and villi, related to Figure 4. (A) Venn diagrams showing the overlap between the target loci in crypts and villi for H3K27me3, H3K4me1, H3K4me3 and H3K9me3, respectively.

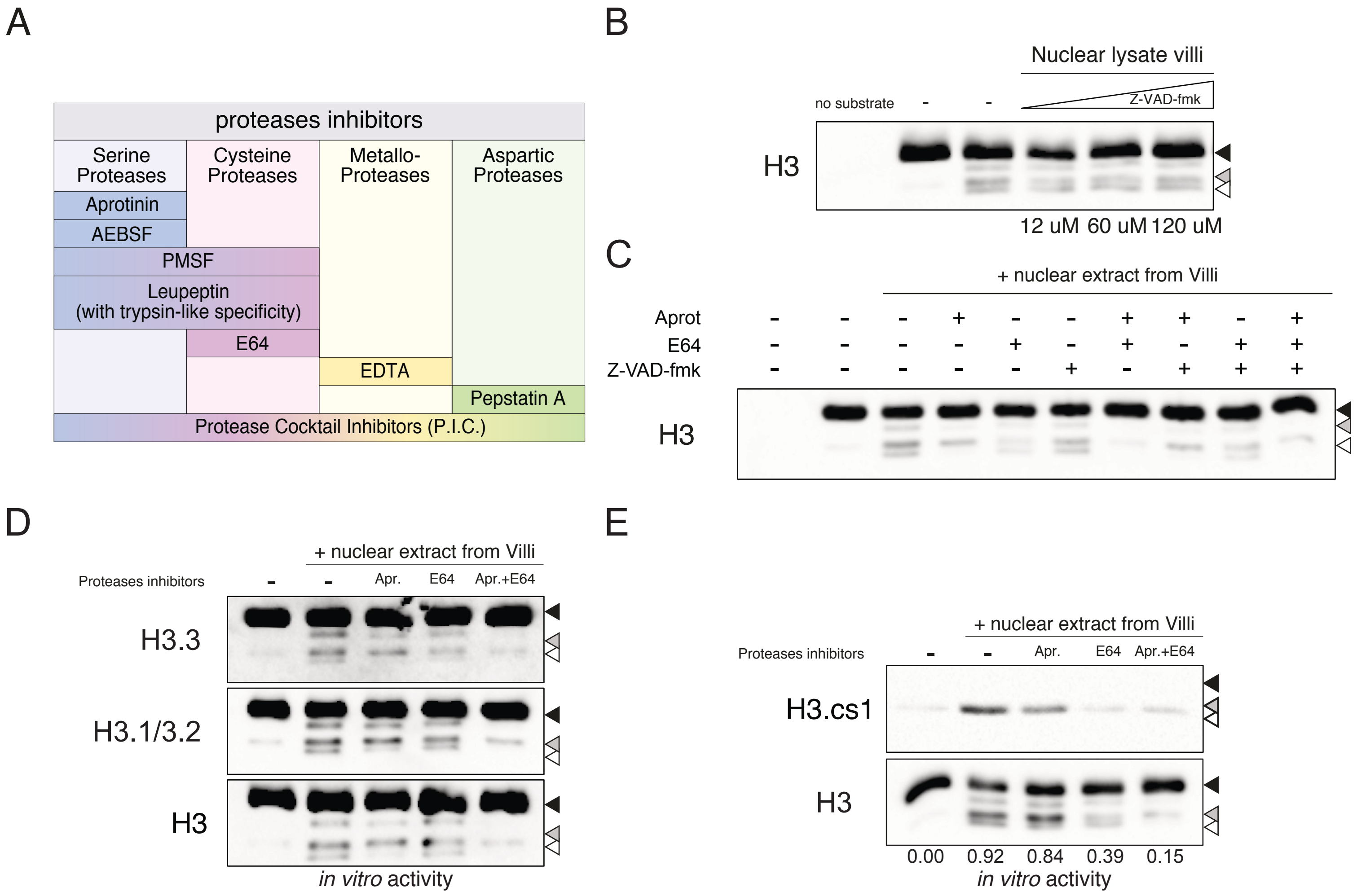

A
proteases inhibitors
Serine Proteases
Cysteine Proteases
Metallo-Proteases
Aspartic Proteases
Aprotinin
AEBSF
PMSF
Leupeptin (with trypsin-like specificity)
E64
EDTA
Pepstatin A
Protease Cocktail Inhibitors (P.I.C.)
B
Nuclear lysate villi
no substrate
Z-VAD-fmk
H3
12 uM 60 uM 120 uM
C
+ nuclear extract from Villi
Aprot
E64
Z-VAD-fmk
H3
D
+ nuclear extract from Villi
Proteases inhibitors
Apr.
E64
Apr.+E64
H3.3
H3.1/3.2
H3
in vitro activity
E
+ nuclear extract from Villi
Proteases inhibitors
Apr.
E64
Apr.+E64
H3.cs1
H3
0.00 0.92 0.84 0.39 0.15
in vitro activity

Supplementary Figure S4. Cathepsin L is the Cysteine protease involved in histone H3 clipping, related to Figure 5. (A) Table resuming the spectrum of inhibition for the used compounds in Figure 5A. (B) Clipping *in vitro* assay performed on nucleosomes purified from 293T cells using 10 ug of villi nuclear extract with or without increasing amounts of Z-VAD-fmk, pan-caspase inhibitor: 12 uM, 50 uM and 120 uM. (C) Clipping *in vitro* assay performed as in (B) with or without protease inhibitors: Aprotinin (Aprot.), E64, Z-VAD-fmk or a combination of them. (D) Western blot analyses of histone H3 and its variants, H3.1/.2 and H3.3, of clipping *in vitro* assay performed with or without protease inhibitors: Aprotinin (Aprot.), E64 or a combination of these latter (Aprot.+E64). (E) Western blot analyses of histone H3 and H3.cs1 truncation of clipping *in vitro* assay performed with or without protease inhibitors: Aprotinin (Aprot.), E64 or a combination of these latter (Aprot.+E64) with the indicated antibodies, specific for H3 PTMs. Numbers below indicate intensities ratio of clipped vs full length H3 signals as clipping quantification.

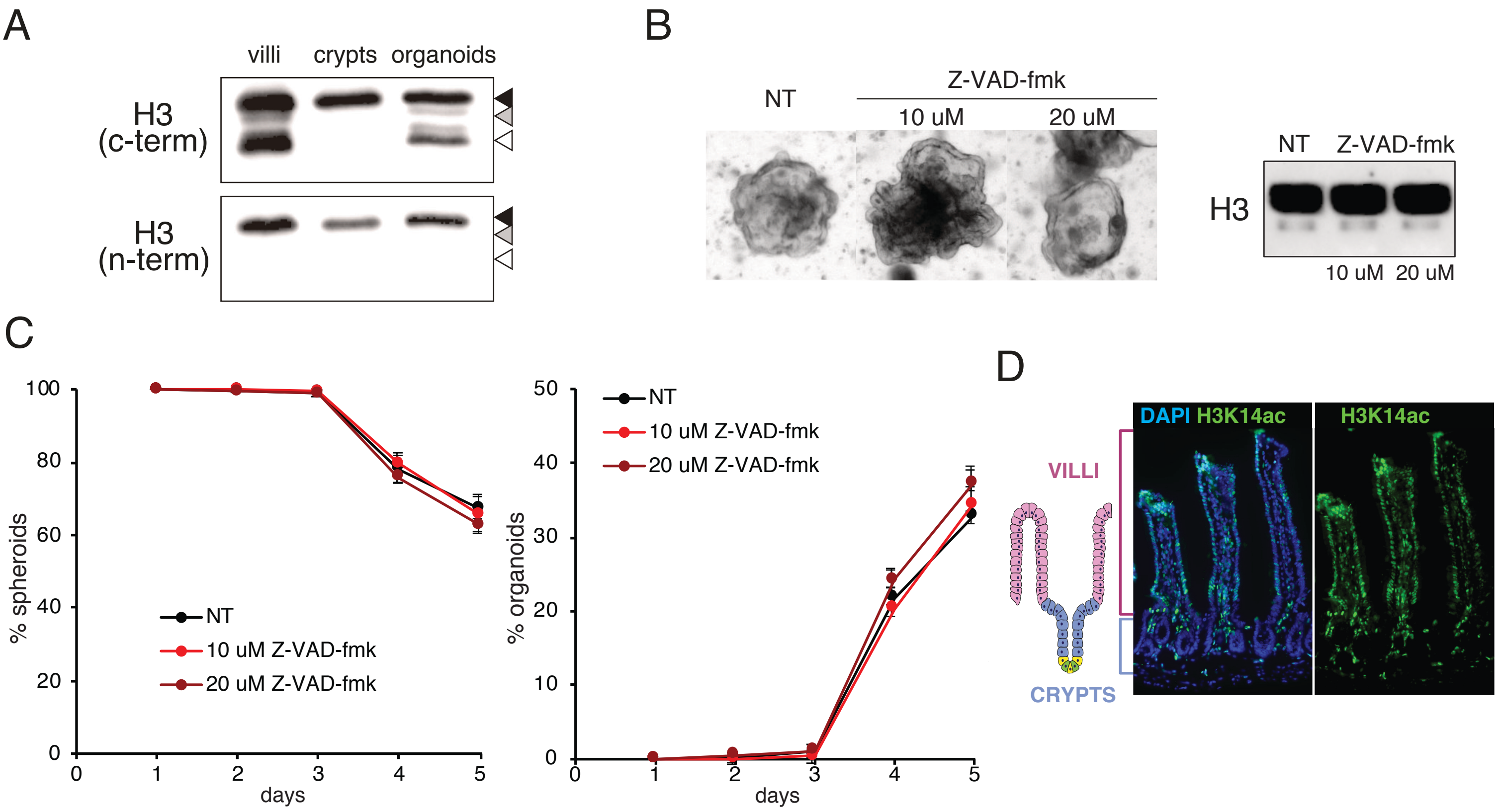
A
villi
crypts
organoids
H3 (c-term)
H3 (n-term)
B
NT
Z-VAD-fmk
10 uM
20 uM
NT
Z-VAD-fmk
H3
10 uM
20 uM
C
% spheroids
NT
10 uM Z-VAD-fmk
20 uM Z-VAD-fmk
days
% organoids
NT
10 uM Z-VAD-fmk
20 uM Z-VAD-fmk
days
D
VILLI
CRYPTS
DAPI H3K14ac
H3K14ac

Supplementary Figure S5. Histone H3 clipping is involved with intestinal organoids maturation, related to Figure 7. (A) Western blot analysis of histone H3 deriving from villi, crypts or organoids using antibodies raised against opposite extremities of H3 (C-terminus and N-terminus). (B) Pictures at 5 days of growth and Western blot analysis of histone H3 extracted from organoids cultured for 6 days in absence (Not Treated, NT) or in presence of pan caspase inhibitor (Z-VAD-fmk, 10 uM and 20 uM). (C) % spheroids and % organoids observed in the three conditions of the experiment in (B). (D) Immunofluorescence staining of H3K14ac (green) in the duodenum epithelial tissue. The DAPI (blue) staining represents the nucleus (magnification 40x).

Supplementary Table S1.

AUC values measured for all the H3 samples analyzed

Supplementary Table S2.

List of antibodies and compounds used in this study
